# Characterization of adipose-derived stromal/stem cells from the twitcher mouse model of krabbe disease

**DOI:** 10.1186/1471-2121-14-20

**Published:** 2013-04-16

**Authors:** Xiujuan Zhang, Julie A Semon, Shijia Zhang, Amy L Strong, Brittni A Scruggs, Jeffrey M Gimble, Bruce A Bunnell

**Affiliations:** 1 Center for Stem Cell Research and Regenerative Medicine, Tulane University School of Medicine, New Orleans, LA 70112, USA; 2 Department of Pharmacology, Tulane University School of Medicine, New Orleans, LA 70112, USA; 3 Stem Cell Biology Laboratory, Pennington Biomedical Research Center, Baton Rouge, LA 70808, USA; 4 Division of Regenerative Medicine, Tulane National Primate Research Center, Covington, LA 70433, USA

**Keywords:** Adipose stem cells, ASCs, Krabbe disease, Twitcher mice, Autologous transplantation

## Abstract

**Background:**

Krabbe disease, also known as globoid cell leukodystrophy, is an autosomal recessive neurodegenerative disease caused by the genetic deficiency of galactocerebrosidase (GALC), a lysosomal enzyme responsible for the degradation of several glycosphingolipids like psychosine and galactosylceramide. In order to investigate whether GALC deficiency in Krabbe disease affects adipose-derived stromal/stem cell (ASC) properties and if the ASCs could be used as a source of autologous stem cell therapy for patients with Krabbe disease, ASCs isolated from subcutaneous adipose tissue of Twitcher mice (a murine model of Krabbe disease) and their normal wild type littermates were cultured, expanded, and characterized for their cell morphology, surface antigen expression, osteogenic and adipogenic differentiation, colony forming units, growth kinetics, and immune regulatory capacities *in vitro*.

**Results:**

ASCs from Twitcher mice (TwiASCs), when compared to ASCs from normal mice (WtASCs), have a reduced osteogenic differentiation potential, have less self-replicating and proliferative capacity, although they have the same fibroblast morphologies and cell sizes. However, surprisingly, the TwiASCs demonstrated similar immune-suppressive capacities as their counterparts WtASCs did when they were transwell co-cultured with macrophages *in vitro*.

**Conclusion:**

This study reveals that Twitcher ASCs exhibit differences in the biologic potential when compared to their counterparts from normal mice. The changes in Twitcher ASCs may be influenced by the GALC deficiency in Twitcher mice. Nevertheless, none of the changes preclude the use of the TwiASCs for autologous applications.

## Background

Krabbe disease, also known as globoid cell leukodystrophy, is an autosomal recessive neurodegenerative lysosomal storage disorder affecting both the central nervous system (CNS) and peripheral nervous system (PNS) [1]. The disease is caused by the genetic deficiency of galactocerebrosidase (GALC), a lysosomal enzyme responsible for the degradation of several glycosphingolipids like psychosine and galactosylceramide [1-4]. The accumulation of GALC substrates, especially psychosine, leads to progressive demyelination of axons and death of oligodendrocytes in the CNS and Schwann cells in the PNS [5].

Based on the age of onset and severity, Krabbe disease is clinically divided into infantile type, juvenile type and adult type [6]. The infantile Krabbe disease is most common with onset between 3–6 months of age of symptoms including vomiting, difficulty in feeding, extreme irritability, complete blindness, deafness, spastic paralysis, extreme emaciation and dementia, leading to death by 2 years of age [6-10]. Currently, there is no cure for Krabbe disease. The only approved clinical therapies for Krabbe disease are bone marrow transplantation (BMT) and umbilical cord blood transplantation (UCB), after myeloablative chemotherapy, and such a procedure has only been beneficial in the infantile type if it was performed before the onset of clinical symptoms [7,11]. However, the majority of pre-symptomatic infants receiving transplantation have been reported to develop severe disabilities, including motor and language deterioration [11-13]. Moreover, BMT and UCB therapies are limited by the scarcity of donors, and can be complicated by the development of graft versus host disease (GVHD), a serious and often fatal condition resulting from the immunological attack of foreign T cells against the patient [14]. One study had reported that GVHD developed in 8 out of 11 asymptomatic and 5 out of 14 symptomatic Krabbe disease patients treated with UCB [11].

Adipose-derived stromal/stem cells (ASCs) can be isolated from lipoaspirate following liposuction. ASCs have the potential to differentiate into adipocytes, osteocytes, chondrocytes, myocytes and neuronal cells when stimulated with appropriate induction factors [15-21]. ASCs have received increasing attention from both biological scientists and clinicians because of the cells’ ability to differentiate into multiple lineages, the abundance and easy accessibility of adipose tissues, and the feasibility of harvest by a minimally invasive procedure. In addition, ASCs have been reported to reduce inflammation [22,23] and secrete growth, angiogenic and anti-apoptotic factors [24,25]. Therefore, ASCs from individual Krabbe disease patients may be a source of mesenchymal stromal/stem cells for autologous cell therapy. Autologous transplantation has lower rates of post-transplant complications and infections compared to allogeneic transplantation. Moreover, there are no concerns about donor availability and risk of histoincompatibility in autologous transplantation. Autologous ASCs offer numerous advantages from regulatory, histocompatibility, and immunological perspectives [26].

The Twitcher mouse model was developed through spontaneous mutation of the GALC gene at the Jackson Laboratory in 1976 [8,27,28] and shares many neuropathologic findings with the human infantile type of Krabbe disease. In this study, ASCs harvested from Twitcher mice (TwiASCs) and normal wild type mice (WtASCs) from the same litters were culture expanded, characterized, and compared based on features such as differentiation potential, colony forming unit capabilities, cell surface marker profiles, growth kinetics and cytokine expression.

## Results

### Morphology and cell size of TwiASCs and WtASCs

**Figure 1:**
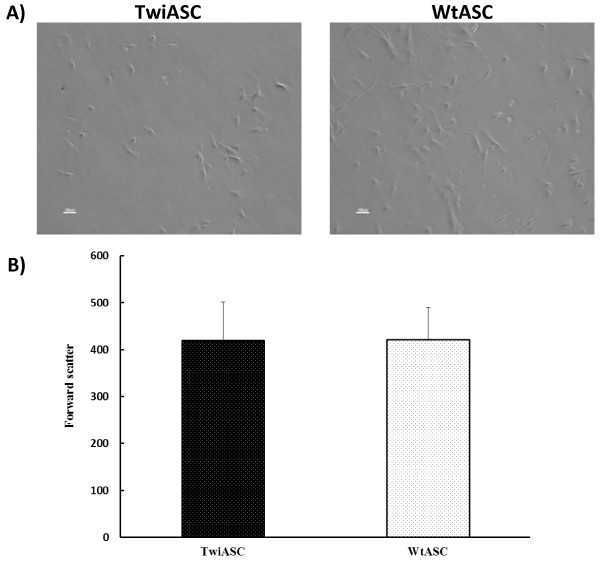
**Morphology and cell size of TwiASCs and WtASCs. A**) Morphology of TwiASCs and WtASCs. Both cell types retain the thin spindle fibroblast-like morphology described for ASCs. **B**) Cell size based on forward scatter signal of flow cytometry (*P*=0.96, *t*-test, n≥10).

The cell morphology of TwiASCs was consistent with that of the WtASCs, which were fibroblast-like in appearance (Figure 1A). The sizes of both cell types were also analyzed based on the forward scatter signals of flow cytometry. As shown in Figure 1B, the cell size of TwiASCs versus WtASCs was not significantly different (*t*-test, *P*>0.05).

### Flow cytometric analysis of surface antigens on TwiASCs and WtASCs

**Figure 2:**
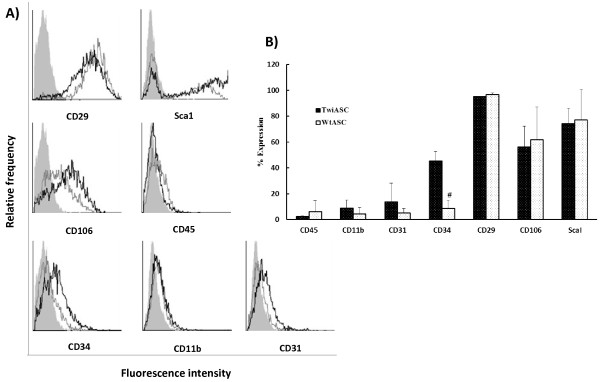
**Cell surface phenotype analysis of TwiASCs and WtASCs. A**) Cells were analyzed by flow cytometry for MSC surface markers CD29, Sca1 and CD106; hematopoietic markers CD34 and CD45; phagocytic lineage marker CD11b; and endothelial marker CD31. Gray filled: isotype control; gray line: WtASCs; black line: TwiASCs. **B**) The percentage expression of each surface marker. All cells were analyzed at passage 3. # means *P*< 0.05 *vs* TwiASCs for CD34 (*t*-test, n=3).

ASCs from Twitcher mice and normal wild type mice were isolated, cultured, expanded, and analyzed for surface markers by flow cytometry at passage 3. ASC surface markers CD29, Sca1, and CD106; hematopoietic markers CD34 and CD45; phagocytic lineage marker CD11b; and endothelial marker CD31 were tested. As shown in Figure 2, both TwiASCs and WtASCs were positive for CD29, Sca1, and CD106. Both cell types are negative for CD45, CD11b, and CD31. However, there is a significant difference in CD34 expression between TwiASCs and WtASCs (*t*-test, *P*<0.05). The ASCs were harvested from three Twitcher mice and three wild type mice respectively, and both TwiASCs and WtASCs were repeated three times independently by flow cytometry and similar results were obtained in all studies.

### Differentiation assays for TwiASCs and WtASCs

**Figure 3:**
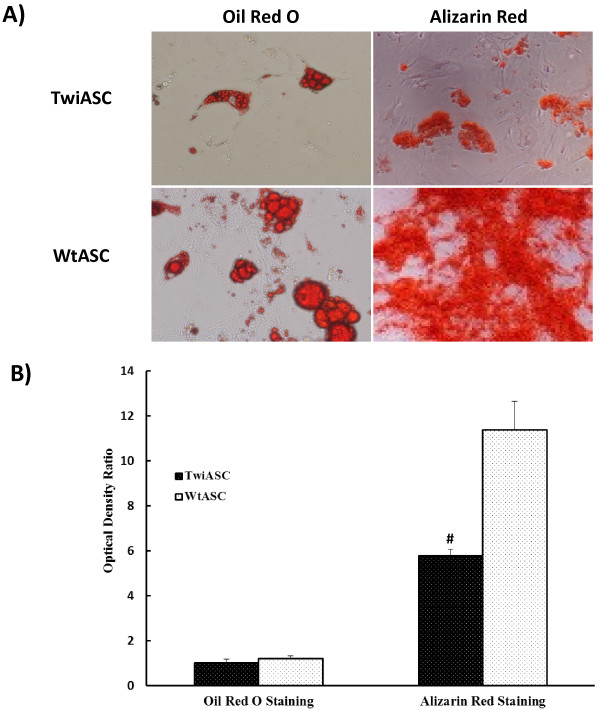
**Qualitative and quantitative differentiation of TwiASCs and WtASCs along osteogenic and adipogenic lineages. A**) Image of differentiated TwiASCs and WtASCs. Passage 3 TwiASCs and WtASCs were incubated in adipogenic or osteogenic differentiation media for 21 days and stained, respectively, with Oil Red O and Alizarin Red. For osteogenic differentiation, images were collected at 4× magnification. For adipogenic differentiation, images were obtained at 10× magnification. **B**) Quantification of osteogenesis and adipogenesis. For the quantitation of osteogenesis, the cells were de-stained with 10% cetylpyridinium chloride after stained with Alizarin Red. For the quantitation of adipogenesis, the cells were de-stained with isopropanol. Optical density (OD) was measured at 584 nm and normalized to protein content. The bar graph represents the ratio of normalized OD of differentiated cells and normalized OD of control cells. # means *P*< 0.05 *vs* WtASCs (*t*-test, n=3).

Twitcher ASCs and wild type ASCs were cultured in osteogenic and adipogenic differentiation medium for 3 weeks to test their lineage differentiation efficiency. On day 21, the ASCs were stained with Alizarin Red to assess bone mineralization, and with fresh Oil Red O for lipid droplets. Both TwiASCs and WtASCs efficiently differentiated into osteocytes and adipocytes (Figure 3). However, as shown in Figure 3A, the WtASCs appear to have greater differentiation capacity for osteogenic differentiation than the TwiASCs. Quantification of the differentiation levels of both cell types (Figure 3B) further demonstrated that WtASCs (OD ratio=11.39±0.73) could differentiate into osteocytes to a much greater degree than TwiASCs (OD ratio=5.78±0.17) (*t*-test, *P*<0.05) over the period of the assay. Quantification of adipocyte differentiation indicated that WtASCs (OD ratio=1.20±0.07) have a slightly higher capacity than TwiASCs (OD ratio=1.01±0.10), but not statistically significant (*t*-test, *P*>0.05). The graph in Figure 3B represents the ratios of OD of differentiated cells normalized to control cells.

### Osteogenic markers in WtASC and TwiASC

**Figure 4:**
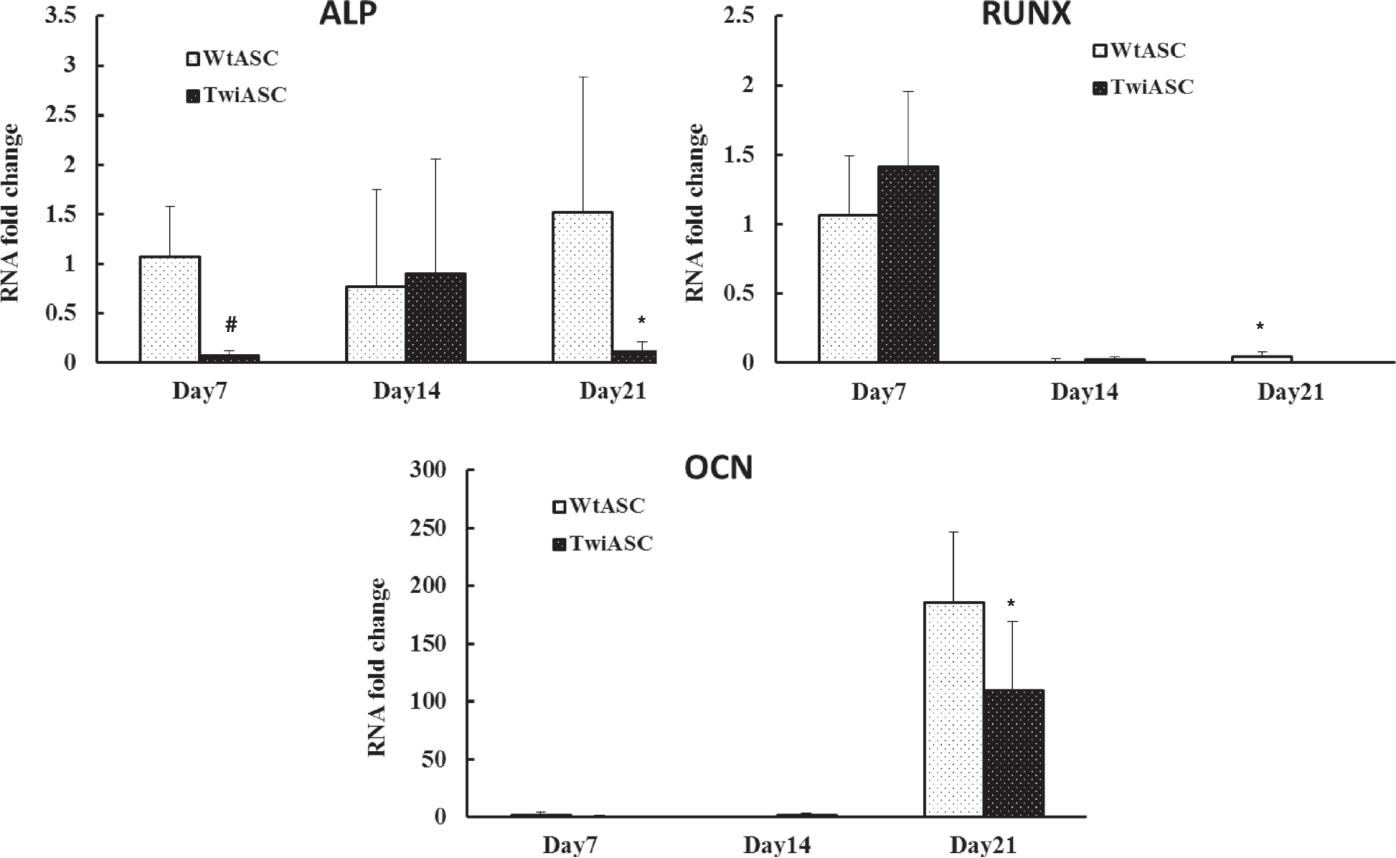
**Real-time PCR analysis of osteogenic lineage marker expression in differentiated TwiASCs and WtASCs.** TwiASCs and WtASCs were induced for osteogenic differentiation. On day 7, 14 and 21, the cells were harvested and total RNA was extracted for PCR analysis of osteogenic markers ALP, RUNX and OCN. # means P< 0.05 vs WtASCs on day 7 and * means P< 0.05 vs WtASCs on day 21 for ALP, RUNX and OCN respectively (*t*-test, n=6).

As shown in Figure 3, WtASCs appear to have greater differentiation capacity for osteogenic differentiation than the TwiASCs. To further investigate their osteogenic potentials, replicate cultures of WtASC and TwiASC were induced to undergo osteogenic differentiation and harvested on day 7, 14 and 21 for alkaline phosphatase (ALP), runt-related transcription factor 2 (RUNX), and osteocalcin (OCN) mRNA analysis by real-time PCR. ALP catalyzes the hydrolysis of monophosphate esters at a high pH, and it is one of the first functional genes expressed in the process of calcification [29]. RUNX is a bone-specific transcription factor, and it stimulates OCN and other bone specific gene transcriptions via the binding of its runt domain to their promoters [30]. OCN, a marker of bone formation, is the most abundant and most widely studied non-collagenous protein in bone [31]. As demonstrated in Figure 4, WtASCs have a much higher ALP mRNA expression than TwiASC on day 7 (*t*-test, *P*<0.05). By day 21, WtASCs demonstrated higher expression in all the three lineage markers than TwiASC (*t*-test, *P<0.05*) (Figure 4).

### Colony forming unit assay

**Figure 5:**
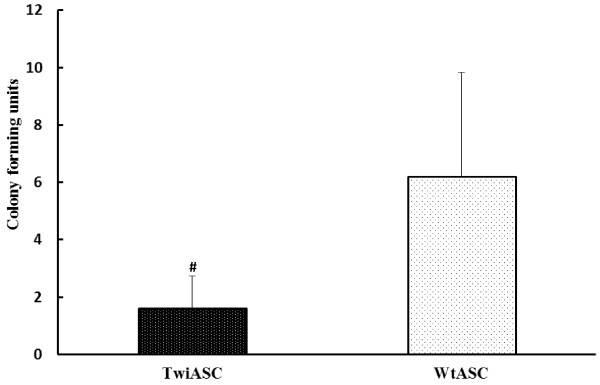
**Colony forming unit assays for TwiASCs and WtASCs.** 100 cells were plated on 56.7 cm^2^ Nunc cell culture plates and incubated for 14 days. Cells were stained with 3% crystal violet, and colonies 2 mm or larger in diameter were counted. # means *P*< 0.05 *vs* WtASCs (*t*-test, n=5).

The colony forming unit (CFU) assay was used to assess the self-renewal ability of the cells. Both TwiASCs and WtASCs were seeded onto 56.7 cm^2^ Nunc cell culture plates at a total of 100 cells per plate and the number of colonies was enumerated after 14 days of culture. The CFU assay results (Figure 5) demonstrated that WtASCs have a significantly higher self-replicating capacity (6.20±1.62) than TwiASCs (1.60±0.51) (*t*-test, *P*<0.05).

### Growth kinetics of TwiASCs and WtASCs

**Figure 6:**
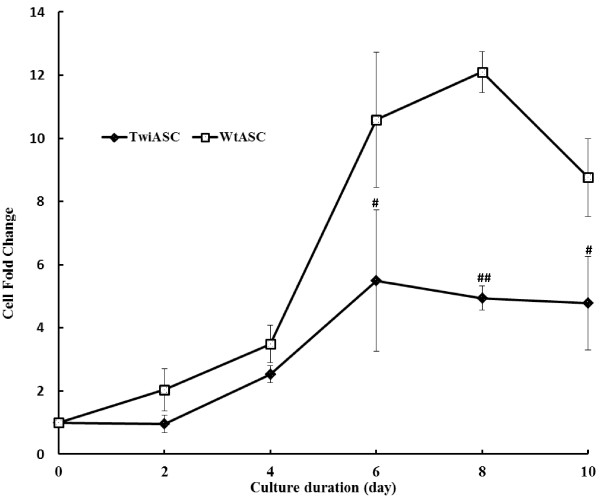
**Growth curve analysis of TwiASCs and WtASCs.** The cells were originally seeded at 500 cells/cm^2^ in 56.7 cm^2^ Nunc cell culture plates and cultured for 10 days. Fold increase in cell number was analyzed every 2 days throughout the culture period and was calculated by comparing the cell number at each time point to the original plating cell number. # means *P*< 0.05 vs WtASCs on day 6 and day 10 respectively, and ## means *P*< 0.001 *vs* WtASCs on day 8 (*t*-test, n=3).

In order to test their proliferative capacity, TwiASCs and WtASCs were plated on 56.7 cm^2^ Nunc cell culture plates at a density of 500 cells/cm^2^ and cultured for 10 days. The fold-increase in cell number was analyzed every 2 days throughout the culture period and was calculated by comparing the cell number at each time point to the original plating numbers. As illustrated in Figure 6, the TwiASCs have a much slower growth rate than WtASCs. For the 10-day culture period, TwiASCs only had a maximal fold-increase highest of 5.49 ±1.29 while WtASCs had a maximum of 12.10 ±0.38. After that, both TwiASCs and WtASCs reached their growth plateau and started to decline.

### Regulation of TwiASCs and WtASCs on inflammatory cytokine expression in primary macrophages

**Figure 7:**
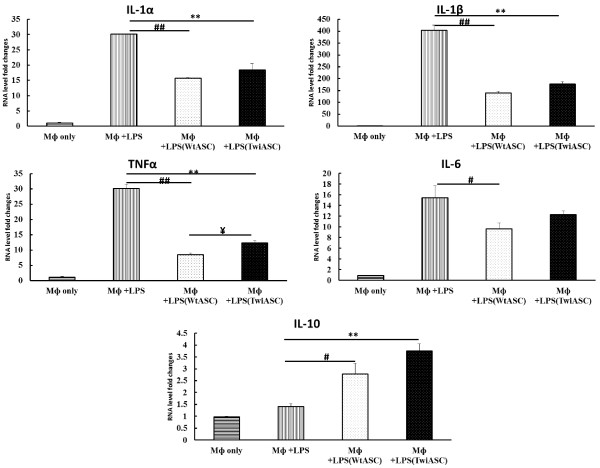
**The inflammatory cytokine profiles of LPS-stimulated macrophages.** The ASCs and thioglycolate-elicited macrophages were transwell co-cultured and incubated for another 24 hours in the presence of 30 ng/ml LPS. The macrophage cells were collected, and total RNA was extracted for real-time PCR quantification of inflammatory cytokines IL-1α, IL-1β, TNFα, IL-6 and IL-10. The macrophage (MФ) alone and LPS stimulated macrophage (MФ+LPS) were used as controls. ## means *P*< 0.01 MФ+LPS vs MФ+LPS (WtASCs) (*t*-test, n=3); **means *P*< 0.01 MФ+LPS vs MФ+LPS (TwiASCs) (*t*-test, n=3); # means *P*< 0.05 MФ+LPS vs MФ+LPS (WtASCs) (*t*-test, n=3); and ¥ means *P*< 0.05 MФ+LPS(WtASCs) vs MФ+LPS (TwiASCs) (*t*-test, n=3).

The thioglycolate-elicited peritoneal primary macrophages were seeded onto 24-well cell culture plates. After 6-hour incubation, 0.4 μm pore size transwells were placed in the 24 well plates and TwiASCs and WtASCs were added on top of each transwell. The transwell co-culture was incubated overnight followed by stimulation of the cells with LPS for an additional 24 hours. The expression levels of inflammatory cytokines, IL-1α, IL-1β, TNFα, IL-6 and IL-10 were analyzed by real-time PCR to determine whether TwiASCs and WtASCs could effectively regulate their expression. As illustrated in Figure 7, little cytokine production was observed in macrophages in the absence of LPS, and LPS stimulation triggered an outburst of cytokine production by macrophages, especially the pro-inflammatory IL-1α, IL-1β, TNFα, IL-6 cytokines. Remarkably, WtASC significantly reduced the macrophage ability to produce IL-1α (*t*-test, *P*<0.01), IL-1β (*t*-test, *P*<0.01), TNFα (*t*-test, *P*<0.01), IL-6 (*t*-test, *P*<0.05), and enhanced the production of the anti-inflammatory cytokine IL-10 (*t*-test, *P*<0.05). TwiASCs demonstrated similar immune-suppressive capacities *in vitro* as WtASCs with the exception that they did not suppress the IL-6 expression by the macrophages (Figure 7, *t*-test, *P*>0.05).

## Discussion

This study compared adipose stem cells from Twitcher mice to those isolated from their wild type littermates in order to determine whether ASCs with the mutation associated with Krabbe disease continue to display normal phenotype. This has relevance to their potential use in future transplantation studies.

From the morphologic perspective, TwiASCs and WtASCs appeared similar with a fibroblast-like morphology and had no significant difference in cell size based on direct observation and on the forward scatter data of flow cyotmetry. Both TwiASCs and WtASCs were analyzed by flow cytometry for specific cell surface markers such as CD29, CD106, Sca1, CD45, CD34, CD11b, and CD31. The cell surface marker profiles for both cell types were comparable with the exception of CD34 expression where there was a significant difference between TwiASCs and WtASCs. The expression of CD34 differed as a function of passage numbers for TwiASCs. It was positive in passage 3 as shown in Figure 2, but gradually diminished its expression along higher passages, which is typical of ASCs in culture (data not shown). There have been reports showing that CD34 is consistently down- regulated while culturing over several passages [24,32], which is consistent with our findings that TwiASCs gradually lost their expression of CD34 over progressive passages while all other surface markers tested remained the same. However, there have been never detectable levels of CD34 expression in WtASCs. CD34 is a surface marker for hematopoietic stem cells, endothelial progenitor cells and satellite cells. The difference of CD34 expression between TwiASCs and WtASCs may be due to differences between the adipocyte niches within adipose depots of Twitcher mice and their wild type littermates.

Both TwiASCs and WtASCs could be readily differentiated into osteocytes and adipocytes. Although the lipid droplets produced by WtASCs were larger, in general, than those of TwiASCs as observed under the microscope, there was no significant difference in the accumulation of neutral lipids between TwiASCs and WtASC. However, WtASCs exhibited an increased differentiation along the osteogenic lineage relative to TwiASCs. The study of osteogenic markers in differentiated WtASC and TwiASC (Figure 4) demonstrated that WtASCs, compared to TwiASCs, have significantly increased levels of expression. ALP is a cell surface glycoprotein that is involved with mineralization and its role in mineralization process occurs during early differentiation. Measurement of increased ALP expression at the mRNA level has been taken as a reliable indication of the osteoblastic phenotype [29,33]. OCN is a matrix protein that regulates osteoclast activity, and is a late marker of differentiation at the mineralization stage [33]. Reduced osteogenic differentiation capacity of TwiASCs correlated with reduced ALP and OCN expression of TwiASCs, although the mechanism(s) regulating the reduced ALP and OCN expression in TwiASCs is unclear. However, it has been noticed that Twitcher mice demonstrated postnatal bone retardation, and Contreras and colleagues [34] demonstrated that Twitcher mice demonstrated osteopenic phenotype, and their bones were much smaller and weighed less compared to normal mice. The same group [34] also reported that the accumulated psychosine induced by GALC-deficiency is one of the potential factors that resulted in the stunted bone growth in Twitcher mice. Psychosine accumulation in TwiASCs may play a role in their reduced differentiation capacity along the osteogenic lineage.

The colony forming unit assay and growth curve analyses were used to compare the self-renewal and proliferative capacities between the TwiASCs and WtASCs. The results clearly indicated that the TwiASCs have a significantly reduced ability to form single-cell-derived colonies when plated at low density in comparison with the WtASCs. Moreover, cultures of TwiASCs seemed to grow much slower than did their wild type counterparts. However, many studies have demonstrated that the therapeutic effects of ASCs are not only solely the results of their capacity for differentiation along mesenchymal and non-mesenchymal lineages, but more importantly related to their immune regulatory capacities [23,35-38]. Macrophages are a key component of the innate immune system and are widely distributed in many different tissues. In addition to their role in initial phases of immune defense, macrophages play an indispensable role in later phases of tissue homeostasis and repair. The interaction of mesenchymal stem cells (MSCs) with macrophages has been widely investigated in recent years [39-42]. Ne’meth et al. [40] demonstrated that MSCs increased the IL-10 production by LPS-stimulated macrophages *in vitro*. They also demonstrated that administration of MSCs improved organ function and reduced mice mortality for the treatment of sepsis, and the therapeutic effects of MSCs were induced upon their stimulation by IL-10 produced by macrophages. In our study, the TwiASCs and WtASCs were co-cultured in transwell plates with peritoneal macrophages stimulated by LPS, and the *in vitro* expression of inflammatory cytokines by macrophages was analyzed by real-time PCR. The results indicated that TwiASCs demonstrated similar immune-suppressive capacities *in vitro* as their counterparts WtASCs. These data suggested that, although TwiASCs had reduced osteogenic differentiation, self-renewal and proliferative capacities, they are quite similar to WtASCs in their anti-inflammatory properties. This is an important finding since the therapeutic effects of adult stem cell therapy had been shown more related to their immune-modulatory capacities.

## Conclusion

ASCs from Twitcher mice, when compared to ASCs from normal mice, have a reduced osteogenic differentiation potential, have less self-replicating and proliferative capacity, although they have the same fibroblast morphology and cell sizes. However, surprisingly, the TwiASCs demonstrated similar immune-suppressive capacities as their counterparts WtASCs did when they were co-cultured with macrophages *in vitro*. These findings suggest that while the GALC deficiency in Twitcher mice does influence ASC profiles in some aspects, it does not affect their immune-suppressive capacities significantly. These outcomes have relevance since the immune-suppressive function is potentially a critical mechanism that would account for the therapeutic benefit of ASC transplantation in Krabbe disease.

## Methods

### Isolation and culture of adipose-derived stem cells

The Twitcher mice and their littermates were obtained from breeding pairs carrying a natural mutation in the GALC gene. Breeding pairs were originally purchased from the Jackson Laboratory (Bar Harbor, ME). All the protocols and experimental procedures were approved by the Institutional Animal Care and Use Committee at Tulane University. Genotyping of Twitcher mice was performed on post-natal day 14 by real time PCR modified as previous described [43]. The subcutaneous fat pads were isolated from the post-natal day 34 moribund Twitcher mice and their wild type littermates, rinsed with Hank’s balanced salt solution (HBSS) (Life Technologies, Grand Island, NY) to remove blood and hair contamination, and digested with 0.1% collagenase type 1 solution (Life Technologies) for approximately 4 hours at 37°C under vigorous agitation. The digested adipose tissue samples were then filtered through a 70-μm nylon mesh cell strainer (BD Biosciences, Bedford, MA), and centrifuged at 500×*g* for 10 minutes at room temperature (RT). The pellets were re-suspended and cultured in the growth medium DMEM: F12 (Life Technologies) supplemented with 10% fetal bovine serum (FBS) (Atlanta Biologicals, Atlanta, GA), 2 mM L-glutamine (Life Technologies), and 1% antibiotic/antimycotic (Penicillin/streptomycin/amphotericin, Life Technologies).

### Flow cytometry

The following antibodies were used to define the surface markers expressed by the ASCs: CD29-FITC (fluorescein isothiocyanate), CD34-FITC, CD106-PE (vascular cell adhesion molecule-1 (VCAM-1)), CD31-PE (phycoerythrin), CD45-PE, CD11-PE, and Sca1-PE (stem cell antigen-1). All of the antibodies were purchased from BD Biosciences. The ASCs were cultured to 70% confluence, trypsinized, pelleted, and re-suspended in 500 μl phosphate buffered saline (PBS) (Life Technologies). The cells were incubated with the antibodies for 30 minutes at RT, then washed with PBS, and analyzed by Cytomics FC500 (Beckman Coulter, Brea, CA). The results were analyzed with CXP analysis software (Beckman Coulter).

### Colony forming unit assay

ASCs at passage 3 were seeded onto 56.7 cm^2^ Nunc cell culture plates (Nalge Nunc International, Rochester, NY) in 5 replicates at a total of 100 cells per plate. Growth media was changed every 3–4 days. After 14 days, the cells were washed with PBS, stained with 3% crystal violet in 100% methanol for 30 minutes at RT, and then washed with deionized (DI) water at least 3 times to remove excess dye. All colonies greater than 2 mm in diameter were counted.

### Differentiation

ASCs at passage 3 were seeded with a density of 6×10^4^ cells/well on 6-well Nunc plates (Nalge Nunc International) and cultured to approximately 90% confluence before adipogenic and osteogenic differentiation media were added. Adipogenic differentiation medium was ASC growth medium supplemented with 5 μg/ml insulin, 50 μM indomethacin, 1 μM dexamethasone and 0.5 μM 3-isobutyl-1-methylxanthine (all media supplements were purchased from Sigma, St Louis, MO). Osteogenic differentiation medium was ASC growth medium supplemented with 1nM dexamethasone, 20 mM β-glycerolphosphate, 50 μM L-ascorbic acid 2-phosphate sesquimagnesium salt, and 50 ng/ml L-thyroxine sodium pentahydrate. Media were changed twice per week for 3 weeks. For adipogenic differentiation, the cells were washed with PBS, fixed with 10% formalin (Sigma) for 20 minutes at RT, washed again with PBS, stained with Oil Red-O (Sigma) for 20 minutes at RT, washed again with PBS until wash was clear. For the detection of osteogenesis, the cells were washed with PBS, fixed with 10% formalin for 20 minutes at RT, washed again with DI water, stained with Alizarin Red (Sigma) for 20 minutes at RT, washed again with DI water until wash was clear. Images were acquired at 10× for adipogenic differentiation and 4× for osteogenic differentiation on Nikon Eclipse TE200 (Melville, NY) with Nikon Digital Camera DXM1200F using the Nikon ACT-1 software version 2.7.

The *levels of adipogenic and osteogenic differentiation were also quantitated. For the quantitation of adipogenic differentiation, the accumulated lipids were eluted with isopropanol after images were captured. The amount of Oil Red O was measured by recording the optical density (OD) of the solution at 584 nm. The results were normalized to the protein content of the samples with the BCA assay (Thermo Scientific, Rockford, IL). For the quantitative osteogenesis assay, the cells were de-stained, after images were taken, with 10% cetylpyridinium chloride (Sigma) for 30 minutes at RT. The amount of Alizarin Red was determined by measuring the OD of the solution at 584 nm. The results were normalized to the protein content of the samples.

### Real-time PCR analysis of osteogenic markers in WtASC and TwiASC

Total RNA was extracted from differentiated WtASC and TwiASC on day 7, day 14, and day 21 using RNeasy Mini Kit (Qiagen, Valencia, CA). RNA concentration and purity were assessed by Nanodrop 2000C spectrophotometer (Thermo Scientific). First strand cDNA syntheses were performed using iScript cDNA Synthesis kit (Biorad, Hercules, CA) after RNA was first treated with DNase (Life Technologies). Primers for mouse alkaline phosphatase (ALP), Runx-2 (RUNX), and osteocalcin (OCN) were commercially synthesized (IDTDNA, Coralville, Iowa), and the sequence of the primers was as previously described [44]. The housekeeping gene β-actin (UniGene: Mm328431) was used as internal reference. The PCR reactions were performed using CFX96 Real Time System (Biorad) in a total final volume of 25 μl containing 12.5 μl SYBR green supermix (Biorad), 1 μl forward primer and 1 μl reverse primer (400 nM), and 1 μl template (500 ng). Reaction mixtures were incubated at 95°C for 3 minutes, and reactions were allowed to proceed via 45 cycles of melting at 95°C for 15 seconds, annealing and extension at 57°C for 30 seconds. Quantification was calculated using ΔΔCt method [44].

### Growth curve

ASCs at passage 3 were seeded onto 56.7 cm^2^ Nunc cell culture plates in triplicate with a seeding density of 500 cells/cm^2^. Growth medium was changed every 3–4 days. Every 2 days for a total of 10 days, the cells were trypsinized, pelleted, re-suspended in 500 μl PBS, and counted using a Countess® Automated Cell Counter (Life Technologies) to analyze the fold increase in cell number.

### Macrophage and ASCs co-culture

Primary macrophages were obtained through thioglycolate-elicited peritoneal isolation using a slightly modified published method [42]. Briefly, 1 ml 3% (w/v) sterile thioglycolate (Sigma) in DI water was injected intraperitoneally into C57Bl6 mice (Jackson Lab) to elicit peritoneal exudate cells. Four days after thioglycolate injection, cells were harvested by peritoneal lavage using 10 ml PBS, treated with red blood cell lysing buffer (Sigma) to remove the red blood cells, assessed for macrophage purity by flow cytometry using FITC-labeled IgG anti-CD11b (BD Biosciences), and finally plated onto 24-well Nunc cell culture plates (Nalge Nunc International) with 200,000 cells per well in RPMI medium (Life Technologies) supplemented with 10% heat inactivated FBS and 1% penicillin/streptomycin. After 6-hour incubation, the non-adherent cells were removed by vigorous washing with PBS. Then 0.4 μm pore size 24 well transwell inserts (Corning, Lowell, MA) were placed into the 24-well plate, and TwiASCs or WtASCs were plated in each transwell insert with 40,000 cells per well (ASC: macrophage ratio=1:5). Cells were cultured overnight, incubated another 24 hour after the addition of 30 ng/ml lipopolysaccharide (LPS, Sigma), and then total RNA was extracted from the macrophages using RNeasy Mini Kit. RNA concentration and purity were assessed by Nanodrop 2000C spectrophotometer.

### Real-time quantitative PCR

RNA was first treated with DNase, and first strand cDNA syntheses were performed using iScript cDNA Synthesis kit. Real-time PCR assay was performed to analyze the levels of mouse inflammatory cytokines IL-1α, IL-1β, TNFα, IL-6 and IL-10 expressed by macrophages transwell co-cultured with TwiASCs and WtASCs. PCR analyses were performed using CFX96 Real Time System in a total volume of 20 μl containing 10 μl Taqman mastermix (Applied Biosystems, Foster City, CA), 1 μl primer and probe mix (Applied Biosystems), and 2 μl template. Reaction mixtures were incubated at 50°C for 2 minutes and 95°C for 10 minutes, and reactions were allowed to proceed via 40 cycles of melting at 95°C for 15 seconds, annealing and extension at 60°C for 1 minute. The housekeeping gene β-actin (UniGene: Mm328431) was used as internal reference. Quantification was calculated using ΔΔCt method [45]. Controls were RNA from macrophages cultured alone with and without LPS.

### Statistical analysis

Groups of data were analyzed by one-way analysis of variance (ANOVA). For pair-wise comparisons, the F-test was used to determine whether a given pair of population variances was equal (α<0.05). This information was then used in designating the appropriate t-tests (typically heteroscedastic) to perform for comparing the means of population pair, with significance defined as *P*<0.05. All values were reported as mean± SD.

## Competing interests

The authors declare that they have no competing interest.

## Authors’ contributions

XZ conceived of the study, performed all the experiments and drafted the manuscript. JS helped design the study and edited the manuscript. SZ helped the macrophage co-culture experiment. AL helped the differentiation quantification experiment and edited the manuscript. BS did the genotyping of Twitcher mice and edited the manuscript. JG edited the manuscript. BB supervised the entire study and edited the manuscript. All authors read and approved the manuscript.
